# Handheld reflectance confocal microscopy: Personalized and accurate presurgical delineation of lentigo maligna (melanoma)

**DOI:** 10.1002/hed.26545

**Published:** 2020-11-24

**Authors:** Yannick S. Elshot, Biljana Zupan‐Kajcovski, William M. C. Klop, Marcel W. Bekkenk, Marianne B. Crijns, Menno A. de Rie, Alfons J. M. Balm

**Affiliations:** ^1^ Department of Dermatology The Netherlands Cancer Institute Amsterdam the Netherlands; ^2^ Department of Dermatology Amsterdam UMC, University of Amsterdam Amsterdam the Netherlands; ^3^ Department of Head and Neck Oncology and Surgery The Netherlands Cancer Institute Amsterdam the Netherlands; ^4^ Department of Oral and Maxillofacial Surgery Amsterdam UMC, University of Amsterdam Amsterdam the Netherlands

**Keywords:** diagnostic accuracy, lentigo maligna, presurgical mapping, reflectance confocal microscopy

## Abstract

**Background:**

The surgical treatment of lentigo maligna melanoma is associated with high rates of local recurrence. Handheld reflectance confocal microscopy (HH‐RCM) allows for in vivo presurgical detection of subclinical lentigo maligna (melanoma) (LM/LMM).

**Methods:**

A single‐center retrospective study from December 2015 to July 2017. Frequency and extent of negative surgical margins, and the diagnostic accuracy of presurgical mapping by HH‐RCM was determined.

**Results:**

Twenty‐six consecutive patients with LM/LMM were included. In 45.8%, HH‐RCM detected subclinical LM with a sensitivity of 0.90 and specificity of 0.86. The management was changed in two (7.7%) patients. Of the 24 remaining lesions, 95.8% were excised with negative margins with a mean histological margin of 3.1 and 5.3 mm for LM and LMM, respectively. At a mean follow‐up of 36.7 months, there was one (4.8%) confirmed recurrence.

**Conclusions:**

Our method of presurgical delineation by HH‐RCM appears to provide a reliable method for the surgical treatment of LM/LMM with a limited rate of overtreatment.

## 1 INTRODUCTION

Lentigo maligna (LM) is an in situ melanoma with a predilection for the head and neck.^1^ The treatment of LM is aimed at preventing progression into lentigo maligna melanoma (LMM). While the risk of progression is low, the incidence of both LM and LMM are currently on the rise.^2^, ^3^

The treatment of LM/LMM is associated with high rates of local recurrence^4^ due to the frequent subclinical spread of atypical melanocytes.^1^, ^5^ The optimal approach to surgical treatment thus remains a topic of significant debate, especially given the presentation of the lesion in a cosmetically and functionally sensitive area. While international guidelines currently recommend surgical margins of 5 mm for LM, this has been reported to be insufficient in up to 62.7% of cases, with rates of recurrence ranging from 6% to 20%.^6^

A dermatoscope is a handheld device that allows the magnification and visualization of skin morphology that is not visible to the naked eye and is widely used by dermatologists in the diagnosis of pigmented lesions, including LM/LMM.^7^ Even so, the usefulness of dermatoscopy for detecting LM beyond the clinical margin remains limited, as it seems unable to detect individual atypical melanocytes at the lesion's periphery.^8^ To minimize tissue excision while still achieving local control, several surgical techniques are used, including Mohs micrographic surgery (MMS) and staged excision.^6^ While MMS allows for a complete (100%) assessment of the surgical margin, it requires extensive training. Moreover, it is not universally accepted in the treatment of melanocytic lesions because frozen sections can result in artifactual as well as fixational changes in excision specimens. This issue can be circumvented by using rushed permanent paraffin embedded sections or staged excision techniques in which the entire peripheral margin is assessed in several stages without the need of special training or equipment. While offering a definite advantage over conventional excision in achieving local control, these techniques remain time‐intensive.

Reflectance confocal microscopy (RCM) is a noninvasive imaging technique that allows for in vivo visualization of cutaneous structures at the cellular level, up to the level of the papillary dermis. Past studies have shown that RCM is well suited for identifying subclinical disease beyond the margin delineated using dermatoscopy.^9^, ^10^, ^11^ In a prospective study using traditional arm‐mounted RCM (AM‐RCM), the resulting surgical area was 40% larger on average than when determined by dermatoscopy alone.^11^ More recently, a handheld RCM (HH‐RCM) device consisting of a smaller, non‐fixated probe was introduced. This flexibility allows for more rapid evaluation and access to the more concave areas in the head and neck.

For this retrospective study, we developed a new method for in vivo presurgical delineation of LM/LMM in the head and neck using HH‐RCM‐assisted conventional excision.

## 2 MATERIALS AND METHODS

Consecutive patients with histopathological confirmed LM or LMM seen at the Netherlands Cancer Institute (NKI) from December 2015 to July 2017 were eligible for inclusion. All cases were identified through the local Tumor Registration Database. Inclusion criteria consisted of (a) primary/recurrent LM or LMM ≤ T2 classification according to the 7th edition of the American Joint Committee on Cancer (AJCC) guidelines, (b) localization in the head and neck, and (c) HH‐RCM‐assisted surgery. Patients with (a) T3‐4 classification LMM (AJCC 7th edition), (b) receiving diagnostic excisions (2 mm surgical margin), or (c) nonsurgical treatment were excluded. According to standard of care in the NKI, all patients were assessed by both a board‐certified dermatologist as well as a head and neck surgeon. All histological slides revised by experienced melanoma dermatopathologists. For invasive LMM, routine workup included ultrasound examination, followed by fine needle aspiration cytology (FNAC) in case of suspected macroscopic lymph nodes metastases. For cT1b or higher classified melanomas, sentinel lymph node biopsy (SNB) was discussed with the patient in case of negative ultrasound and/or FNAC. All SNBs were performed simultaneously with the HH‐RCM‐assisted WLE. Confocal imaging and analyses were performed by a single investigator (Yannick S. Elshot), who built up 14 months (1 d/wk) of experience with HH‐RCM before embarking on this study. The study protocol was performed in accordance with the ethical guidelines of the 1975 Declaration of Helsinki. Following a review by the NKI Institutional Review Board, the approval of the ethics committee was waived.

### 2.1 Presurgical mapping procedure

According to standard of care, the clinical border was identified using noncontact polarized dermatoscopy (DermLite DL4, 3Gen, Inc, San Juan Capistrano, California), followed by delineation of the surgical margin with a margin of 5 or 10 mm according to the 7th edition of the AJCC guidelines.^12^

Handheld RCM imaging was performed using the commercially available VivaScope 3000 device (CaliberID, Henrietta, New York; MAVIG GmbH, Munich, Germany). Surgical treatment was performed directly following the HH‐RCM imaging and analyses. Therefore, the investigator (Yannick S. Elshot) was blinded to the histopathological outcome. Prior to margin delineation, the central area of the lesion was assessed to determine the predominant architecture of the lesion. If there was suspicion of invasive melanoma, the lesion was excised with a 2 mm margin for appropriate staging.

**FIGURE 1:**
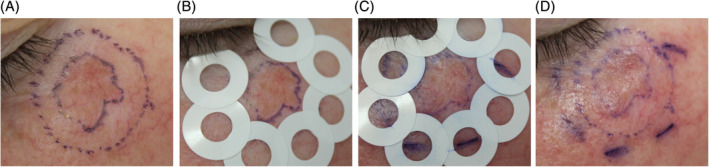
A, Step 1: Lentigo maligna located on the left zygoma delineated by a surgical marker including the 5 mm surgical margins. B, Step 2: Circumferential placement of adhesive rings (5.5 mm diameter and 4 mm inner area) bordering the surgical margin. C, Step 3: Evaluation of the inner areas of the adhesive rings. In the presence of lentigo maligna criteria a new margin was drawn, and the margin persevered in the absence of subclinical lentigo maligna. D, Step 4: New adhesive rings were placed at the new margins and steps 1 to 3 repeated until the entire circumference was negative for lentigo maligna criteria on HH‐RCM examination [Color figure can be viewed at wileyonlinelibrary.com]

**FIGURE 2:**
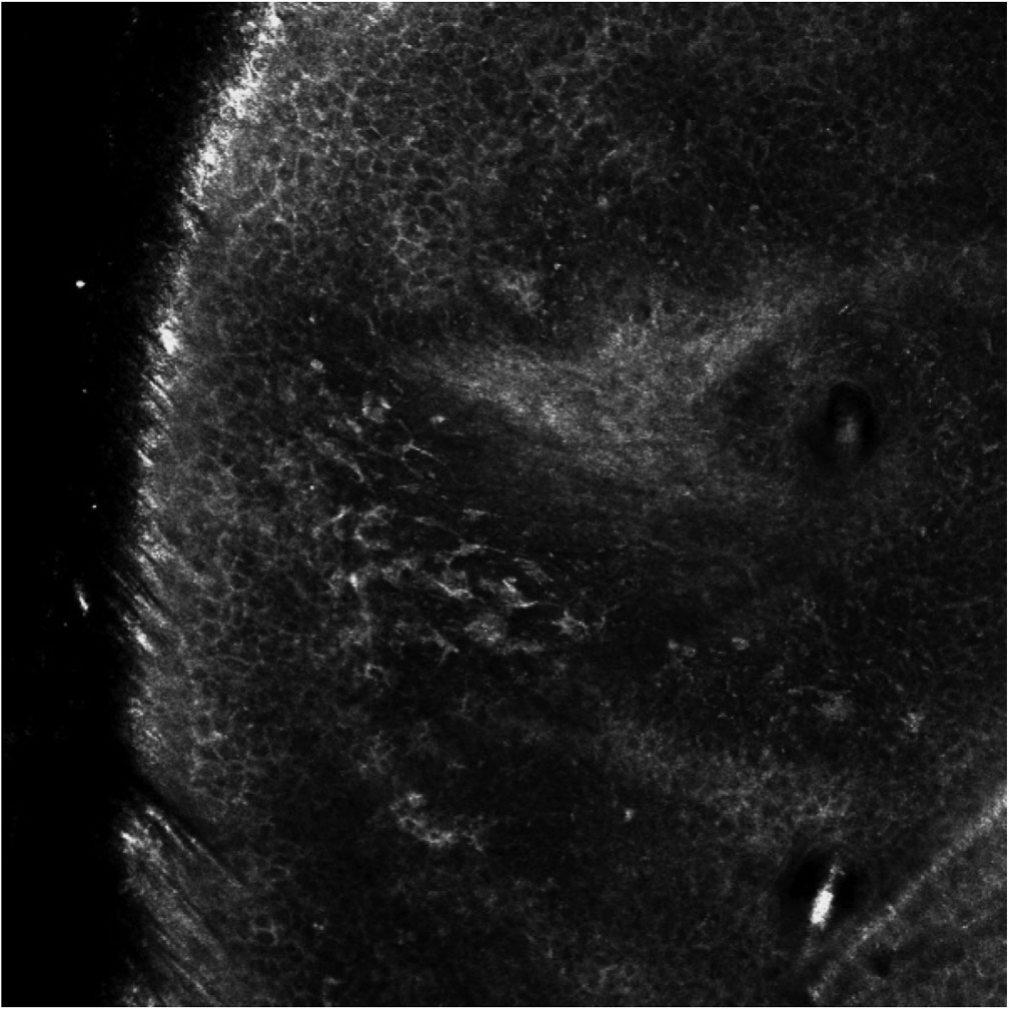
Handheld reflectance confocal microscopy of the inner area of an adhesive ring showing atypical dendritic cells at the level of the dermo‐epidermal junction

All mapping procedures were performed using commercially available circular adhesive rings, according to the following procedure^13^ (Figure 1A‐D):

1. Adhesive rings (inner diameter 5.5 mm; border width 4 mm) were applied, overlapping and bordering the entire circumference of the outer margin.
2. Inner areas of the adhesive rings were examined. The margin was considered positive for LM/LMM if epidermal round large/pleomorphic pagetoid cells, follicular or dermo‐epidermal junction localization of atypical cells (round or dendritic) were present (Figure 2). The margin was redrawn depending on the extension from the previous margin (ie, 1‐2 fields of view at the midline and in case of 2+ fields of view at the distal inner border).
3. New adhesive rings were placed at the new margins, and the process repeated until negative margins were achieved.
4. In case of the absence of LM/LMM the margin was preserved.

Clinical characteristics were recorded, including age and sex, localization, extent of pigmentation, and histopathological diagnosis.

Histological margin assessment was considered the reference standard. All histological examinations were performed by experienced melanoma pathologists blinded to the RCM margin delineation outcomes. Excisions were fixated in 4% formaldehyde overnight, followed by ink application to secure the resection borders. Two ink colors were applied to the resection margins to divide the long axis of the excision into two halves. Thereafter, 3‐mm‐thick slides were cut perpendicular to the long axis resulting in a sequence of slices that all contained epidermis with lesion in relation to the colored 3‐ and 9‐hour skin resection margin and the deep dermal/subcutaneous resection margin. The 12‐ and 6‐hour tops were prepared separately in order to identify potential spread along the long axis and where necessary, deeper cuts of the tops in the direction of the resection margin were performed until no lesions was visible anymore. When the deepest cut of the top was still positive, the resection margin at that site was considered positive. Next, material was paraffin‐embedded, stained by standard hematoxylin and eosin staining and, where necessary, additional immunohistochemistry for melan‐A, sox10, or S100 to stain melanocytes was applied. Histological data included the histopathological diagnose, margin status, extent of the free margins, and Breslow thickness.

### 2.2 Statistical analysis

The statistical analysis was performed using SPSS software version 22.0 (SPSS Inc, Chicago, Illinois). Absolute and relative frequencies were described for all study characteristics. The diagnostic outcome of HH‐RCM was evaluated by assessing the frequency and extent (mm) of negative margins as reported in the final pathology report. In addition, a 2 × 2 contingency table (Table 2) was created to evaluate the accuracy of our method in detecting subclinical disease beyond the surgical margin, compared with the histopathological margin outcomes. Histological margins were deemed acceptable as ≤5 and ≤10 mm for LM and LMM, respectively. Following these parameters, larger histological margins were considered surgical overtreatment. The outcomes of diagnostic accuracy were defined as follows: (a) true positive, subclinical LM on HH‐RCM with acceptable histological margins; (b) false positive, subclinical LM on HH‐RCM with surgical overtreatment; (c) true negative, no subclinical LM on HH‐RCM and negative histological margins; and (d) false negative, (no) subclinical LM on HH‐RCM and positive histological margins.

## 3 RESULTS

**FIGURE 3:**
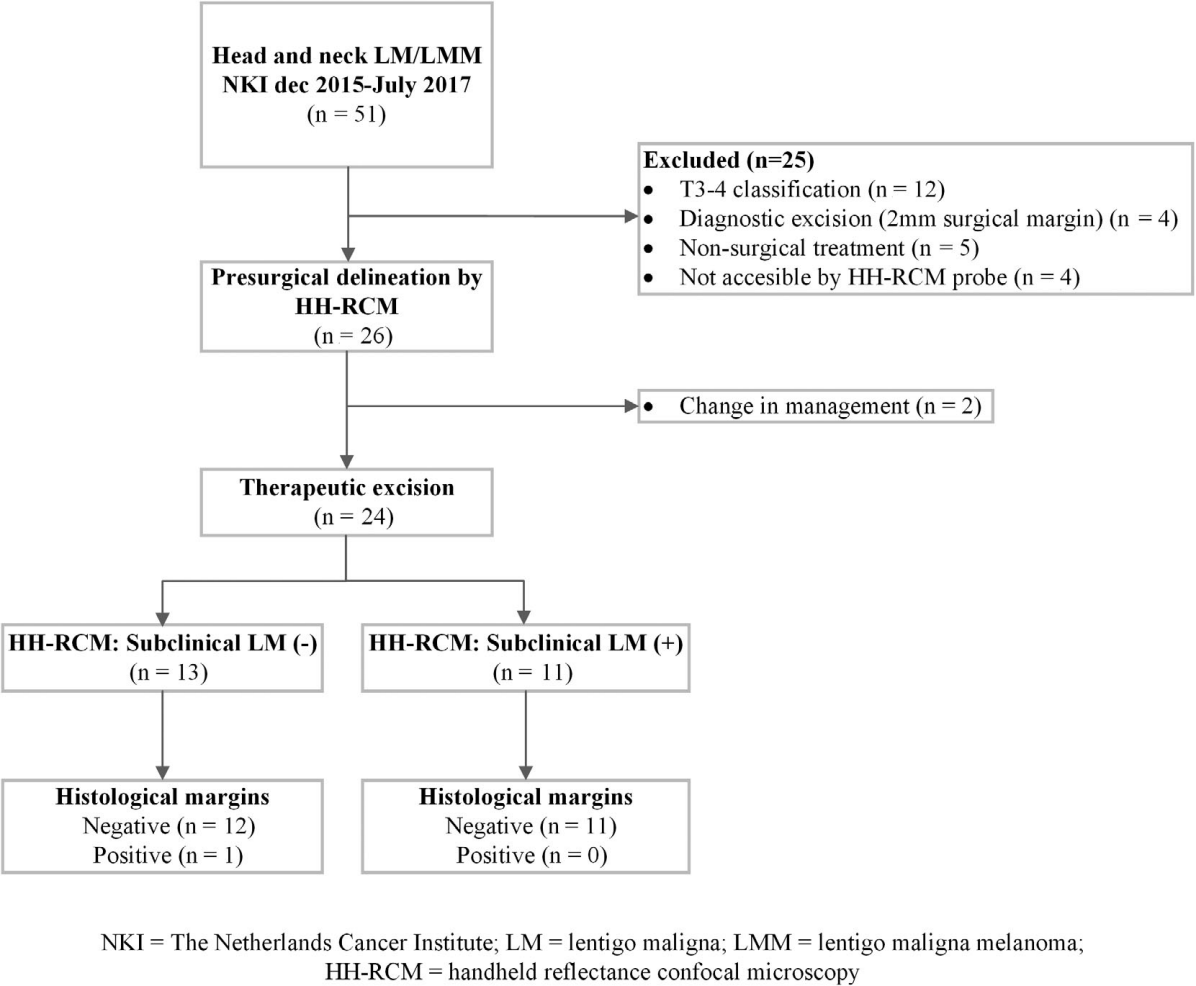
Patient flowchart

Between December 2015 and July 2017, 51 consecutive patients with histopathological confirmed LM/LMM in the head and neck were diagnosed in the NKI (Figure 3). The clinical characteristics of the 26 included patients are shown in Table 1. Twenty‐one (41.2%) patients met the exclusion criteria, consisting of T3/T4 classified LMM (n = 12; 41.2%), diagnostic excision prior to wide local excision (n = 4) and nonsurgical treatment (n = 5). In addition, 4 (7.8%) patients had lesions not accessible by HH‐RCM (n = 4) so were not included for further analysis.

**TABLE 1:** Clinical data of included lesions

Characteristic	Descriptive data, no. (%)	
Patient characteristics		
Age (SD, range) years	69.5 ± 10.1 (48‐90)	
Sex		
Male	9 (34.6%)	
Female	17 (65.4%)	
Lesion characteristics		
Anatomic localization		
Cheek	12 (46.2%)	
Periorbital	5 (19.2%)	
Scalp	3 (11.5%)	
Nasolabial	2 (7.7%)	
Other^a^	4 (15.4%)	
Pigmentation^b^		
Lightly pigmented	12 (46.2%)	
Pigmented	11 (42.3%)	
Amelanotic^c^	3 (11.5%)	
Diagnostic modality		
Punch biopsy (3 mm)	21 (80.8%)	
Incisional biopsy	1 (3.8%)	
Excisional biopsy	4 (15.4%)	
Histological diagnose	Primary	Recurrent
LM	14 (53.9%)	6 (23.1%)
LMM (Breslow mean; range) (0.6 mm; 0.1‐1.2)	3 (11.5%)	3 (11.5%)

Abbreviations: LM, lentigo maligna; LMM, lentigo maligna melanoma.

^a^ Nose/forehead/ear/neck.

^b^ According to Menzies et al.^26^

^c^ Defined as an erythematous macule/patch.

Following HH‐RCM imaging, management was changed based on the RCM evaluation in two patients. In the first patient, subclinical LM extended more than 10 mm into the skin of the lower eyelid. The subclinical extension was confirmed by a 3 mm punch biopsy and the patient was consequently treated with topical imiquimod. In the second patient, cerebriform nests were seen during RCM examination, which is a rare but highly specific RCM structure correlating with invasive melanoma.^14^ Following a diagnostic excision (2 mm margin) the lesion was upstaged to pT1a LMM (Breslow thickness 0.5 mm without ulceration/dermal mitoses).

The remaining 24 evaluable lesions were excised following the presurgical mapping by HH‐RCM, and consisted of 18 (75.0%) LM, and 6 (25.0%) LMM with a median (IQR) Breslow thickness of 0.5 mm (0.1‐0.5). The LMM consisted of pT1a (n = 5) and pT2a (n = 1) melanoma according to the 7th AJCC staging. The median (IQR) diameter of the included lesions was 19.0 mm (12.3‐32.3).

**FIGURE 4:**
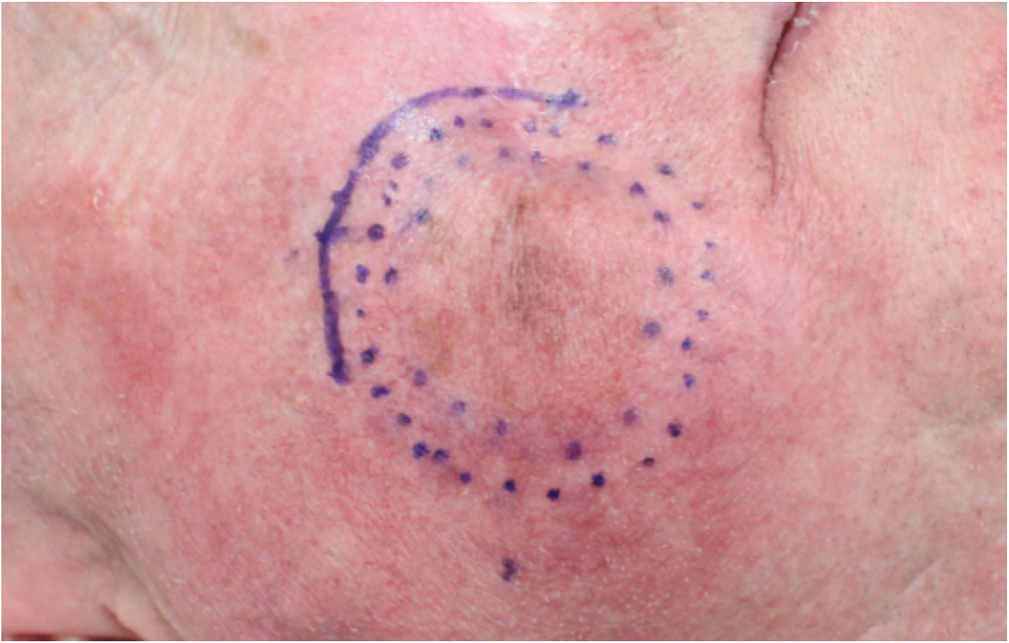
A case of a lentigo maligna on the right cheek with subclinical spread (solid line) [Color figure can be viewed at wileyonlinelibrary.com]

Overall, 95.8% (23 of 24) of the patients had negative margins following excision, including all primary lesions 100.0% (all of 15) and 88.9% (8 of 9) of recurrent lesions. HH‐RCM detected subclinical LM beyond the guidelines‐recommended margin (Figure 4) in 45.8% (11 of 24) of the lesions; however, in 9.1% (1 of 11) of these cases HH‐RCM did not detect the full extent to result in negative histological margins. In the remaining lesions (13 of 24), no subclinical LM was detected by HH‐RCM. However, in 1 of these 13 cases (7.7%) the HH‐RCM outcome was considered a false negative, resulting in positive histological margins (Table 2). This was the only case (4.2%) with positive surgical margins and concerned a patient with recurrent LM.

**TABLE 2:** Subclinical LM detection by HH‐RCM beyond the surgical margins compared to the histological margin outcome (reference standard)

	Histology +	Histology −	
HH‐RCM +	9 (37.5%) (TP)	2 (8.3%) (FP)	11 (45.8%)
HH‐RCM −	1 (4.2%) (FN)	12 (50.0%) (TN)	13 (54.2%)
	10 (41.7%)	14 (58.3%)	

*Note:* Positive (+) outcome defined by subclinical LM on HH‐RCM and histological margins ≤0‐5/10 mm (TP) or positive (FN). Negative (−) outcomes were defined by no subclinical LM on HH‐RCM and histological margins >5/10 mm (FP) or negative margins (TN).

Abbreviations: LM, lentigo maligna; HH‐RCM, handheld reflectance confocal microscopy.

The median (SD; range) of histological margins were 3.3 mm (±2; 0‐7) and 5.3 mm (±3.4; 1‐10) for LM and LMM, respectively. For LMM, the mean (SD; range) histological margin for the invasive component was 9.0 mm (±2.0; 7‐12).

The overall accuracy of subclinical disease detection (Table 3) was 87.5% (95% CI [67.4‐97.3]), with a sensitivity of 0.90 (95% CI [0.55‐1.00]), specificity of 0.86 (95% CI [0.57‐0.98]), and a PPV and NPV of 0.82 (0.55‐0.94) and 0.92 (0.65‐0.99), respectively. For primary lesions, the sensitivity was 1.00 (95% CI [0.48‐1.00]), with a specificity of 0.80 (95% CI [0.44‐0.97]).

**TABLE 3:** Diagnostic accuracy of subclinical LM detection by HH‐RCM

Lesion type	Sensitivity (95% CI)	Specificity (95% CI)	PPV (95% CI)	NPV (95% CI)	Accuracy (95% CI)
All (n = 24)	0.90 (0.55‐1.00)	0.86 (0.57‐0.98)	0.82 (0.55‐0.94)	0.92 (0.65‐0.99)	87.5% (67.4‐97.3)
Primary (n = 15)	1.00 (0.48‐1.00)	80.00 (0.44‐0.97)	0.71 (0.42‐0.90)	1.00	86.7% (59.4‐98.3)
Recurrent (n = 9)	0.80 (0.28‐0.99)	1.00 (0.40‐1.00)	1.00	0.80 (0.40‐0.96)	89.9% (51.7‐99.7)

Abbreviations: CI, confidence interval; HH‐RCM, handheld reflectance confocal microscopy; NPV, negative predictive value; PPV, positive predictive value.

Surgical wounds were closed by primary intention (n = 9), full thickness graft (n = 9), split‐skin grafts (n = 5), and a single reconstructive skin flap (n = 1). A single lesion was upstaged to LMM (pT1a; Breslow thickness 0.5 mm without ulceration/dermal mitoses). Furthermore, the Breslow thickness increased in 3 (12.5%) lesions, resulting in a single LMM being upstaged from a pT2a to a pT3a melanoma. No lymph nodes metastases were detected by ultrasound guided FNAC (0 of 5) or SNBs (0 of 2) in any of the patients.

Four (16.6%) patients died during follow‐up due to unrelated causes, with no signs of local recurrence at a median (IQR) follow‐up of 20.5 months (14.5‐37.8). There was one (4.2%) histologically confirmed recurrence at 10 months of follow‐up in the patient with positive margins following surgical treatment. This patient was subsequently treated with topical imiquimod, showing no sign of recurrence at 17 months of follow‐up.

The remaining 19 (79.2%) patients had a mean follow‐up of 36.7 months (range 26‐49) without local recurrence. One patient with LM developed distant metastasis at 21 months of follow‐up, with no prior history of invasive melanoma. All prior histological specimens were retrospectively reviewed without signs of invasive melanoma. The patient was treated with anti‐PD‐1 checkpoint inhibition (Pembrolizumab) and had (stable) complete remission at 38 months of follow‐up.

## 4 DISCUSSION

While dermatoscopy plays a significant role in the diagnosis of LM/LMM, its efficacy is insufficient for full margin control.^7^, ^8^ RCM therefore has the potential to play a role in the (surgical) management of LM/LMM by detecting atypical melanocytes beyond the margins defined by dermatoscopy. In this study, we evaluated the accuracy of presurgical delineation by HH‐RCM in a consecutive series of patients with head and neck LM/LMM, by which negative margins were achieved in 95.8% of the patients, including all primary (n = 18) and amelanotic (n = 3) cases. Our rate of negative histological margins compares favorably to several other successful approaches using RCM in the surgical treatment of LM/LMM.^10^, ^15^, ^16^ Champin et al used HH‐RCM‐assisted staged excision (ie, Spaghetti technique) of LM/LMM using two operators and were able to identify subclinical LM beyond the guidelines‐recommended margins in all included lesions.^16^ Following the first surgical stage, negative margins were achieved in 85% of the cases. In a follow‐up study, Couty et al were able to fully excise 88% of the lesions after one surgical stage with no reported local recurrences at an average of 44 months of follow‐up.^10^

As we detected subclinical LM in 45.8% of our patients, these patients would most likely have had positive surgical margins following excision in a setting without HH‐RCM. This number is comparable to published data where the guidelines‐recommended surgical margins as being insufficient in up to 24% to 45% of LM treated by wide local excision.^6^ Two lesions were upstaged to invasive melanoma following surgical excision, highlighting the need for caution when opting for nonsurgical treatment modalities. Surprisingly, one out of the four patients who developed distant metastasis had no prior history of invasive melanoma. It is striking that this patient had been surgically treated 6 times before being referred to our center. It is therefore not illogical in this case to assume that an invasive component might have been missed during histological assessment. However, an unknown primary with complete regression cannot be excluded as well.

While HH‐RCM device allows for faster evaluation, one of the disadvantages is the lack of standardized imaging, which makes it more observer and experience dependent. Yélamos et al tried to overcome this limitation by converting videos into larger images using a custom‐made algorithm.^17^ The estimated surgical margins by HH‐RCM were an average of 0.76 mm smaller than the actual surgical margins. Pellacani et al also used video assessment, using superficial epidermal cuts as a reference point during imaging.^16^ With this technique, they were able to achieve negative margins in 93% of the lesions. The video assessment of the margins had a fair/moderate inter‐rater agreement, with a sensitivity and specificity of 92% and 57%, respectively. While highly accurate and reproducible, the epidermal cuts are semi‐invasive, so one must consider the fact that the current commercially available HH‐RCM device does not allow for adequate post‐treatment sterilization without modifications.

Given the above facts, despite of the lack of standardized imaging, HH‐RCM thus seems to be a reliable alternative to traditional AM‐RCM. Nonetheless, in the current study we were unable to determine the inter‐rater reliability because a single investigator performed all the imaging and analysis.

Due to the localization in the head and neck, limiting surgical overtreatment of LM/LMM should also be considered. In two cases (8.3%), HH‐RCM overestimated the extent of subclinical LM. Dendritic cells are a potential source of false‐positive outcomes, as both melanocytic hyperplasia and Langerhans cells can result in hyper‐reflective dendritic cells on RCM.^17^, ^18^ To our knowledge, only one other study has reported on overestimation of the surgical margin using HH‐RCM, which was found in 9.8% of lesion quadrants.^17^ Nevertheless, the reported risk of overtreatment by RCM seems limited. Guitera et al performed 185 punch biopsies prior to excision of LM/LMM.^11^ By using traditional AM‐RCM, the dermatoscopic false negative rate was brought down from 65.0% to 8.3%, with a comparable false positive rate for both techniques (2.4% and 3.2%, respectively). However, compared to HH‐RCM, the use of AM‐RCM is increasingly time‐consuming in larger lesions due to the need of fixation to the skin on several consecutive areas.

There are several limitations in our study, namely the limited sample size. Ideally, only primary LM/LMM should be included, with the aim of preventing local recurrences. On the other hand, the inclusion of more complex cases from a tertiary oncologic referral hospital could have led to an underestimation of our results.

Prior to mapping by HH‐RCM, the lesions were delineated by dermatoscopy. While the use of dermoscopy could be considered standard of care for dermatologists, it could have led to a source of possible bias in our results. The importance of the use of dermatoscopy is shown in a study by Robinson who found that in all 26 cases the total delineated surface area by visual inspection was significantly less than compared to dermatoscopic delineation.^8^ Nonetheless, even though dermatoscopy improves the delineation of LM/LMM compared to the naked eye, it still results in a general underestimation leading to incomplete excisions.

Many surgical techniques have been used to achieve margin control in excised LM/LMM.^19^, ^20^ We chose to combine the presurgical mapping of LM/LMM by HH‐RCM with conventional excision to provide a time‐saving alternative compared to staged‐excision or MMS. This immediately highlights a possible disadvantage of our technique, as it is comparable to in vivo mapping with punch biopsies. Consequently, we did not evaluate 100% of the histological margins, which in turn could have led to an overestimation of the negative margin rate. Nevertheless, with a mean follow‐up of 36.7 months (range 26‐49) and only one local recurrence, the results seem to indicate that using HH‐RCM is a good alternative to staged excision.

Finally, even though the low rate of recurrence in our study is comparable to rates documented in other studies, our follow‐up period was indeed limited. In fact, a recent study showed that at least 4 to 5 years of follow‐up is needed to detect all LM/LMM recurrences following surgical treatment.^21^ Accordingly, to confirm the definitive accuracy of our method, a longer period of follow‐up will be needed in the future.

## 5 CONCLUSION

To our knowledge, this is the first study that attempts to objectify the accuracy of detecting subclinical LM by HH‐RCM by evaluating the histological margins while also remaining mindful of potential overtreatment. Compared to other approaches, we feel the strength of our method is in its reproducibility, fully noninvasive use of RCM, and performance by a single operator. We believe that the low rate of overtreatment is acceptable, and as such, RCM could help to define potentially tissue‐sparing surgical margins in the head and neck. Furthermore, HH‐RCM can be used as an alternative to time‐consuming staged excisions with potentially several stages of permanent section processing. Moreover, due to HH‐RCM findings, the management of 8.3% (n = 2) of the patients was changed. In a study by Guitera et al, 43% of the patients were treated nonsurgically following RCM examination.^11^ Combined with the fact other that studies have also shown RCM to be a valuable tool in the follow‐up of nonsurgically treated LM,^22^, ^23^, ^24^ RCM could potentially play a role in both personalized surgical and nonsurgical management of LM/LMM.^25^

## Data Availability

The data that support the findings of this study are available from the corresponding author upon reasonable request.
